# Review of national research ethics regulations and guidelines in Middle Eastern Arab countries

**DOI:** 10.1186/1472-6939-13-34

**Published:** 2012-12-12

**Authors:** Ghiath Alahmad, Mohammad Al-Jumah, Kris Dierickx

**Affiliations:** 1 King Abdullah International Medical Research Center, King Saud bin Abdulaziz University for Health Sciences, Box: 22490, Riyadh 11426, Saudi Arabia; 2 Centre for Biomedical Ethics and Law, Faculty of Medicine KU Leuven, Kapucijnenvoer 35 Box 7001, Leuven B-3000, Belgium

## Abstract

**Background:**

Research ethics guidelines are essential for conducting medical research. Recently, numerous attempts have been made to establish national clinical research documents in the countries of the Middle East. This article analyzes these documents.

**Methods:**

Thirteen Arab countries in the Middle East were explored for available national codes, regulations, and guidelines concerning research ethics, and 10 documents from eight countries were found. We studied these documents, considering the ethical principles stated in the Declaration of Helsinki, the Council for International Organizations of Medical Sciences (CIOMS) guidelines, and the International Conference of Harmonization - Guidelines for Good Clinical Practice (ICH-GCP). Our paper comprises a complete list of protections, such as confidentiality, informed consent, ethics committees, and others.

**Results:**

This study found different levels and kinds of research ethics regulations and guidelines in the countries examined. Two groups can be distinguished: the countries in the first group have one or more research ethics regulations or guidelines, while the countries in the second group have not yet established any. Most of the documents showed various degrees of deficiencies in regard to ethical protection. The majority of the documents that were examined refer to one or more international documents on biomedical research ethics.

**Conclusions:**

Recently, a lot of efforts have been made in many countries in the Middle East. However, compared with international documents, most of the research ethics documents in use in this region demonstrate numerous deficiencies. As it relates to these documents, extensive differences could be observed in regard to development, structure, content, and reference to international guidelines.

## Background

In recent decades, there has been increased awareness of ethical issues because of the extensive expansion of clinical research and trials. There has also been growing debate over the ethical challenges faced by different societies. Accordingly, scholars have begun to consider how to offer protection to the research community, resulting in the creation of research ethics guidelines in many countries [1].

Ethical guidelines did not truly appear until after World War II. Prior to this point, efforts to regulate human experimentation—such as the codes of Thomas Percival in England (1794)—were few and exceptional [1]. The code of William Beaumont, issued in 1833, is the oldest American document dealing with the ethics of human experimentation [2]. In 1847, the American Medical Association (AMA) issued its code of ethics (latest version, 2001) [2]. However, the well-known Nuremberg Code, issued in 1947, was the actual beginning of established national ethical codes governing medical research [3]. Many countries later issued their own memorandums about research ethics. Nuremberg was followed by the Declaration of Helsinki (adopted, 1964; latest amendment, 2008), established by the World Medical Association (WMA), which created international regulations governing many countries [4]. Many other international guidelines were subsequently proposed by different organizations, including the International Ethical Guidelines for Biomedical Research Involving Human Subjects (1982; revised, 2002) [5], prepared by the Council for International Organizations of Medical Sciences (CIOMS) and the World Health Organization WHO, and the Guidelines for Good Clinical Practice (GCP, 1995; revised, 2004), prepared by the ICH [6]. However, there is a question about the appropriateness of the application of such international guidelines to different regions across the globe, including the Middle East.

Statistics between 2006 and 2010 show a 4 percent rise in the global number of drug trials conducted in the Middle East, which was the largest increase in any region of the world. Conversely, the number of drug trials in North America decreased by 11 percent in the same period [7]. Many factors make the Middle East attractive for clinical research, including its patient diversity, good medical facilities, cost advantages, and favorable infrastructure, especially in the Gulf countries [7]. In addition, recent medical advances and economic growth in many Arab countries have made educational improvement the focus of many governments; thus, many new universities and research centers have appeared, such as the King Saud bin Abdulaziz University for Health Sciences and the King Abdullah International Medical Research Center, which was established in Saudi Arabia in 2005. Moreover, with countries in the Middle East operating under considerably fewer strict ethical guidelines than their European and North American counterparts [7], drug companies are increasingly moving trials of new and untested drugs to Arab countries.

As a result of this increase in clinical research in the Middle East, many ethical issues involving the use of human subjects in these countries have been brought to the attention of ethicists in the region. In a study conducted among members of research ethics committees in Egypt, 92% of interviewees ranked the development of appropriate national guidelines as a major challenge [8]. Moreover, there is a sense that governments and research communities are keen on developing good guidelines for clinical research.

In this paper, we reviewed the relevant guidelines, regulations, and ethical codes of the Arab countries in the Middle East; investigated whether each country had national regulations or guidelines on research ethics; determined whether ethical principles are stated in these guidelines and, if so, which ones; and then compared them with each other and with the major international guidelines.

## Methods

### Data sources and study selection

Using direct access to the websites of various governmental agencies in each country, we conducted a review of the available online direct data sources for guidelines on research ethics and clinical research laws and the indirect data sources included in the codes of ethics. The websites of ministries of health, ministries of higher education, medical councils, the food and drug administrations, research centers, medical unions, and national committees of bioethics in the 13 member nations of the League of Arab States in the Middle East [9]. The countries studied were Kuwait, Qatar, Bahrain, Saudi Arabia, the UAE, Oman, Egypt, Syria, Lebanon, Palestine, Jordan, Iraq, and Yemen.

Our study selection included all national codes or guidelines (after confirmation by professional research ethics experts from each country) available online that addressed research ethics either exclusively or partially. We critically examined all these national codes and guidelines, considering the protections mentioned in the Declaration of Helsinki, the CIOMS guidelines, and the ICH-GCP. Owing to their wide acceptance around the world, we selected the Declaration of Helsinki and the ICH-GCP for comparison. We selected the CIOMS guidelines for the same reason, but also because they were crafted specifically with the aim of applying the Declaration of Helsinki to developing countries in a way that reflects the conditions and needs of biomedical research in those countries, and due to the implications for the multinational or transnational research in which they may be partners [5]. A list of 19 ethical protections taken from these international documents was used in our comparison.

All relevant documents, written in either English or Arabic, were eligible for inclusion.

### Data extraction and synthesis

Our study approach included both qualitative descriptions [10] and comparisons of selected guidelines. First, guidelines from each country were examined for an explicit description of the type of regulations that existed and the entities that released them. We then noted the purpose of the guidelines and sought to ascertain whether the research ethics protocols were determined exclusively by the guidelines.

## Results

We retrieved 10 guidelines from eight different countries. Three of the guidelines came from Saudi Arabia. No national guidelines could be found in the remaining five countries.

### Historical overview

After the establishment of the first international and Western clinical research guidelines, it required a few decades for decision makers in the Arab countries in the Middle East to begin thinking about their own guidelines. The first attempts at crafting clinical research regulations appear as summarized chapters in the general medical ethics guidelines: in Lebanon, the “Law of Medical Ethics” (1994) [11], in Saudi Arabia, the “Ethics of the Medical Profession” (1998; renewed, 2007) [12] and in Egypt, the “Profession Ethics Regulations” (2003) [13].

The Jordanian “Law of Clinical Studies” (2001) [14] was the first effort to regulate, as national law, how clinical research was conducted, and is the first document in the Arab countries to reference international guidelines and regulations such as the Declaration of Helsinki and the ICH-GCP.

Ten years after establishing universal ICH-GCP guidelines (1995), the first local GCPs began to appear in the region through the Saudi Food and Drug Authority’s (SFDA) “Clinical Trial Requirement Guidelines” (2005; renewed, 2008) [15] and the UAE’s “Guidance for Conducting Clinical Trials Based on Drugs/Medical Products & Good Clinical Practice” (2006) [16].

Kuwait’s “Ethical Guidelines for Biomedical Research” (2009) [17] and Qatar’s “Guidelines, Regulations and Policies for Research Involving Human Subjects” (2009) [18] are the first clinical research guidelines in these countries. The Saudi law, “System of Ethics of Research on Living Subjects” (2010) [19], is the most recent document listed and assumes that many regulations, guidelines, and laws are expected to appear in future years.

### Description of the guidelines reviewed

Many differences exist in terms of whether countries in the Middle East have research ethics guidelines. We classified the countries into two main groups. The countries in the first major group already have guidelines and can be divided into two subgroups according to whether they have special national research ethics guidelines (A)—as is the case for Saudi Arabia, Qatar, Bahrain, Kuwait, the UAE, and Jordan—or whether they have general documents that contain some paragraphs about research ethics (B), as in Egypt and Lebanon.

The second major group contains countries that have no special guidelines but either refer to international guidelines, as in Syria [20,21] and Iraq [22] (C), or keep silent (D), as in Oman, Palestine, and Yemen .

Even though the guidelines used in Group A countries have similar names, their structures are not the same. All of them use the words ‘guidelines’ and ‘research’ or ‘trials’ in their titles. Additionally, the UAE’s guideline adds ‘good clinical practice’ to its title (“Guidance for Conducting Clinical Trials Based on Drugs/Medical Products & Good Clinical Practice,” 2006), which seems reasonable because the UAE’s guideline uses the ICH-GCP structure and contains similar chapters, such as a glossary, principles, requirements for the approval of clinical trials, goals, medical institutes, protection of subjects participating in the clinical trial, and responsibilities of the investigator.

This GCP structure is also used by the SFDA guidelines (I in Table 1), which are called the “Clinical Trial Requirement Guidelines” (2005), without mentioning GCP in its title. The Kuwaiti guidelines, “the Ethical Guidelines for Biomedical Research” (2001), contain only a small section that has been adapted from the ICH-GCP, specifically concerning the elements of informed consent.

**Table 1:** Research ethics guidelines found in the 13 countries in the Middle East

		**Country**	**Entity**	**Year**	**Guidelines**
1^st ^Group	A	UAE	Ministry of Health	2006	Guidance for conducting Clinical Trials Based on Drugs/Medical Products & Good Clinical Practice
		Qatar	Ministry of Health	2009	Guidelines, Regulations and Policies for Research Involving Human Subjects
		Bahrain	Ministry of Health	2009	Ethical Guidelines for Health Research
		Kuwait	Kuwait Institute for Medical Specialization (KIMS)	2001	Ethical Guidelines for Biomedical Research
		Saudi Arabia	Saudi Food and Drug Authority (SFDA)	2005/2008	I Clinical Trial Requirement Guidelines
			Saudi Council for Health Specialties	1998/2007	II Ethics of the Medical Professions
			Council of Ministries	2010	III System of ethics of research on living subjects
		Jordan	Prime Minister’s Council	2001	Clinical Research Law. Provisional Law No. (67) for the Year 2001.
	B	Egypt	Ministry of Health & Population	2003	Profession Ethics Regulations No. (238) for the Year 2003.
		Lebanon	Perlman Council	1994	Law of Medical Ethics No. (288) for the Year 1994.
2^nd ^Group	C	Syria	Ministry of Health	-	Refers to Helsinki Declaration, International Ethical Guidelines for Biomedical Research Involving Human Subjects (CIOMS)
		Iraq	Ministry of Health	-	Refers to unknown Guidelines
	D	Oman	-	-	No document
		Palestine	-	-	No document
		Yemen	-	-	No document

The Bahraini (draft 2009) [23] and Qatari (2009) guidelines, in addition to the “Jordanian Law of Clinical Studies” (2001), do not use the ICH-GCP structure and diverge widely from each other. While the Bahraini and Jordanian guidelines are summarized and abstracted, the Qatari document is detailed and has a comprehensive structure (Table 1).

Egypt and Lebanon do not have special guidelines; however, both countries have medical documents containing an abstracted chapter concerning clinical research.

Saudi Arabia is the only country that has three different documents referring to the regulation of research ethics. The first document (I) is a general guide and contains a chapter concerning biomedical research on both humans and animals, referring specifically to the main principles of clinical research. The second document (II) is an official legal document, stated by the council of ministers.

Of the 10 documents related to biomedical research ethics that were found, five are guidelines, three are national laws, and two are medical codes. Two of the three laws (Saudi Arabia and Jordan) focus on medical research, while the third (Lebanon) is a general medical law.

### What ethical protections are mentioned?

Each guideline contains at least two or more protections. The Qatari guideline contains the most protections at 19, followed by the Saudi “Clinical Trial Requirement Guidelines” with 15 protections. The Saudi “Ethics of the Medical Profession,” as well as the Jordanian and Lebanese guidelines come at the end of the list, with only five, three, and two protections, respectively (see Table 2).

**Table 2:** Ethical protections stated in the research ethics document for the 13 countries in the Middle East

	**Frequency of protections**	**Saudi Arabia**			**Kuwait**	**Qatar**	**UAE**	**Bahrain**	**Egypt**	**Lebanon**	**Jordan**
		**SFDA clinical trial requirement guidelines**	**Ethics of medical profession**	**System of ethics of research on living subjects**	**Ethical guidelines for biomedical research**	**Guidelines, regulations and policies for research**	**Guidance for conducting Clinical Trials**	**Ethical guidelines for health research**	**Professional ethics regulations**	**Law of medical ethics**	**Law of clinical studies**
Informed consent	10	+	+	+	+	+	+	+	+	+	+
E. C.	9	+	+	+	+	+	+	+	+	-	+
Scientific validity	9	+	+	+	+	+	+	+	+	-	+
Confidentiality	7	+	-	+	-	+	+	+	+	+	-
Benefits and risks of participation	7	+	+	-	+	+	+	+	+	-	-
Limitations of risk of research on incapables	5	+	-	+	-	+	+	-	+	-	-
Inducement to participate	5	+	+	+	+	+	-	-	-	-	-
Consent of incapables	4	+	-	+	-	+	-	-	+	-	-
Research involving children	4	+	-	+	-	+	-	-	+	-	-
Ethical review of externally sponsored research	3	+	-	-	-	+	-	+	-	-	-
Information in the I. C.	3	+	-	-	+	+	-	-	-	-	-
Who is responsible for collecting I. C.	3	+	-	-	+	+	-	-	-	-	-
Research with limited resources	2	-	-	+	-	+	-	-	-	-	-
Research involving vulnerable persons	2	-	-	-	-	+	-	-	+	-	-
Compensation	2	+	-	-	-	+	-	-	-	-	-
Strengthening ethical and scientific capacity	2	+	-	-	-	+	-	-	-	-	-
Obligation to provide health-care	2	+	-	-	-	+	-	-	-	-	-
Women as research subjects	1	-	-	-	-	+	-	-	-	-	-
Equitable distribution of burdens and benefits	1	-	-	-	-	+	-	-	-	-	-
Total number of protections		15	5	9	7	19	6	6	9	2	3

I. C. = Informed consent, E. C. = Ethics committee.

The obligation to obtain informed consent is at the top of the listed protections and represents the only item mentioned in all of the guidelines. Nine of the 10 guidelines examined mention an obligation of scientific validity and review by an ethics committee, while seven of 10 mention benefits, risks ratios, and confidentiality. Only five guidelines discuss inducements to participate. Items related to incapacitation come next; thus five of 10 guidelines require limiting risks when dealing with incapable people, while only four discuss gaining their consent. Four guidelines specifically mention special protections for research conducted on children. Three or fewer guidelines mention other specific protections. Women and pregnant women are mentioned in only one of the guidelines.

### What external resources are used as reference?

Eight of the guidelines reviewed in our study refer to external resources. Five refer to international guidelines, while the other three claim to respect Islamic law. The Bahraini and Lebanese documents do not refer to any external references. The Declaration of Helsinki is used by five of the guidelines; the ICH-GCP by four, the Nuremberg Code and Belmont report by two, and the CIOMS is used only once. Even though Syria and Iraq do not have national guidelines, the Syrian Ministry of Health refers to the CIOMS guidelines and the ICH-GCP [21], while, on its website, the Iraqi Ministry of Health refers to guidelines extracted from an unknown reference, but mentions these with respect to Islamic law [22] (See Table 3).

**Table 3:** External references mentioned in the research ethics document for the 13 countries in the Middle East

	**Saudi Arabia**			**Kuwait**	**Qatar**	**UAE**	**Bahrain**	**Egypt**	**Lebanon**	**Jordan**	**Syria**	**Iraq**	**Oman**	**Palestine**	**Yemen**
	**SFDA clinical trial requirement guidelines**	**Ethics of medical profession**	**System of ethics of research on living subjects**	**Ethical guidelines for biomedical research**	**Guidelines, regulations and policies for research**	**Guidance for conducting clinical trials**	**Ethical guidelines for health research**	**Professional ethics regulations**	**Law of medical ethics**	**Law of clinical studies**					
Nuremberg code	-	-	-	+	+	-	-	-	-	-	-	-	-	-	-
Belmont report	-	-	-	+	+	-	-	-	-	-	-	-	-	-	-
Declaration of Helsinki	+	-	-	+	+	+	-	-	-	+	-	-	-	-	-
CIOMS Guidelines	-	-	-	-	+	-	-	-	-	-	+	-	-	-	-
ICH-GCP	+	-	-	-	+	+	-	-	-	+	+	-	-	-	-
Others	-	-	-	-	+	-	-	-	-	-	-	+	-	-	-
Islam	-	+	+	-	-	-	-	+	-	-	-	+	-	-	-

## Discussion

### Economic power leads advances

**Figure 1:**
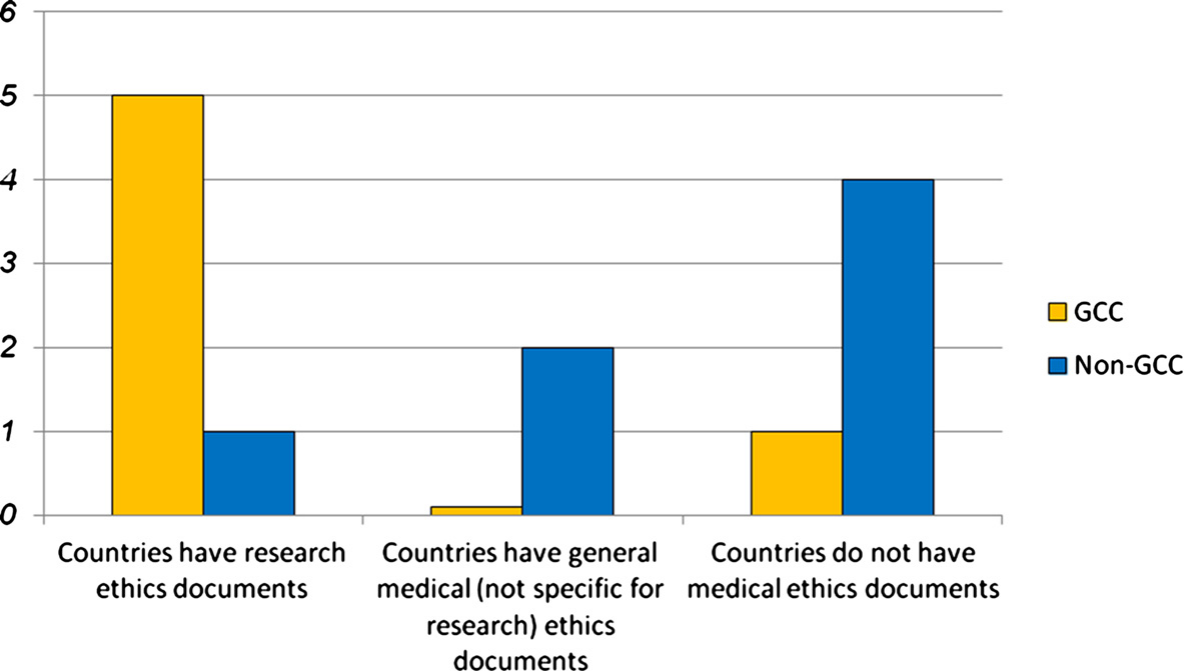
Development of research ethics documents in GCC and non-GCC countries.

Efforts have been made to regulate research ethics in some countries in the Middle East. These efforts can be seen in the new regulations that contain all or most of the protections mentioned in the international guidelines related to research ethics, such as the ICH-GCP, the CIOMS guidelines, and the Declaration of Helsinki. Specifically, these efforts can be seen in the Qatari guidelines and the Saudi “Clinical Trial Requirement Guidelines” crafted by the SFDA, both of which contain the maximum number of protections—19 and 15 respectively. However, the Arab Middle Eastern countries were at different levels with regard to the development of research ethics guidelines; most of the GCC (The Cooperation Council for the Arab States of The Gulf) made advanced steps in this regard compared with non-GCC countries (Figure 1).

The dissimilarities between the countries could be interpreted as resulting from the distinct differences in the levels of financial support awarded to health care and research in GCC and non-GCC countries in the Middle East. Increasing economic power impacts the quality of life in GCC regions, especially in terms of medical services and therapeutic manufacturing and, consequently, also affects medical research and research ethics development (See Figure 1). This is clear when we compare health care expenditure per capita. According to statistics from 2006, expenditure was $538 in Bahrain, $379 in Saudi Arabia, $628 in Kuwait, $716 in the UAE, and $2,151 in Qatar, yet only $103 in Jordan and $38 in Egypt [7]. Expenditure on health in GCC countries enhances the Middle Eastern market for pharmaceuticals. BioPlan Associates expect the health market to be worth approximately $15 billion by 2014 [7].

### Wide variation and major deficiencies

Compared to international guidelines, such as the ICH-GCP and CIOMS, all of the national guidelines described here have many deficiencies in their stated protections, which differ in type and number from one country to another. This variation could be interpreted as a sign of the degree of development of these guidelines on the one hand, or as a sign of the level of importance associated with each protection on the other.

These deficiencies will have an effect on clinical research—especially experimental trials—in the region. Fewer protections mean less strict guidelines, which, in addition to good medical facilities and a suitable research environment, will prove attractive to many pharmaceutical companies who may wish to transfer their research activities to such a region [7]. The many deficiencies observed in these guidelines will leave a question mark as to their adequacy in offering the necessary protections to research subjects in the region.

Scientific validity and obligations for informed consent and review by an ethics committee were mentioned in 10, and nine of the 10 reviewed guidelines, respectively. This indicated the importance of these protections as well as the awareness of the principle of autonomy and freedom in making decisions to participate in clinical research and taking care to carefully review research proposals and concrete ethical guidelines. Confidentiality and the balance of benefits and risks are also important, as they are mentioned seven times. Additionally, even though there is consensus on the guidelines regarding the obligation to have informed consent, only three guidelines described who is responsible for obtaining consent or explained the elements required, which is considered a great deficiency because simply having consent without its elements will invalidate this consent. In the region, there have been few studies about the adequacy of informed consent in medical research; however, this has not yet been reflected in the national document [24,25].

Another clear deficiency in the majority of these guidelines is the failure to reflect on the special societal requirements that result from ignoring vulnerable groups. Only two of the guidelines discussed vulnerable groups, two spoke of individuals with limited resources, four about children, one about women, and one about pregnant women. In fact, it is not only strange, but also a significant deficiency that children were mentioned in only four of the 10 guidelines because the nations in the Middle East are young and children represent a significant portion of their populations. For example, according to statistics from 2007, 32.5% of people in Saudi Arabia are 15 years old or younger [26]. Thus, regulating the participation of children in clinical research and offering them sufficient protections are vital requirements for conducting research in the Middle East.

Despite their importance, providing compensation to research subjects and the equitable distribution of burdens and benefits are rarely among the listed protections. This reflects insensitivity toward these concerns and demonstrates a weakness in the experience and awareness of the results of clinical research. On the other hand, it is, to some extent, understandable that research on populations and communities with limited resources were mentioned in only two guidelines, as the current guidelines that appear in the GCC countries do not really lack resources.

### Are international research ethics guidelines a proper measure for the assessment of research ethics documents in Middle Eastern Arab countries?

The majority of the published and discussed documents refer to one or more international documents on biomedical research ethics, such as the Declaration of Helsinki, ICH-GCP, and others. This also applies to Syria and Iraq, where, until now, no such national documents have been available; however, both countries have referred to one or more of the research ethics international documents on their Ministry of Health websites. This reference raises the question of the appropriateness of using international documents as a standard-bearer for national documents on biomedical research ethics in Middle Eastern countries.

The principles stated in these international documents are undoubtedly universal and reflect shared values. However, bioethics, including research ethics, reflects a mixture of values that derive from religion and culture. It is, consequently, important for national documents to reflect international values as well as the values of their societies. The CIOMS guidelines have significant importance in this regard because they were formulated to be applicable to developing countries [5]. The Islamic reading of this document, as seen by some Muslim scholars assigned by the Islamic Organization of Medical Sciences IOMS, is that the ethical principles of the CIOMS guidelines were fully compatible with Islamic law [27]. Consequently, the CIOMS guidelines can be considered valid for use in the Middle Eastern countries where the majority of the population is Muslim.

Surprisingly, only three documents of the ten studied—two from Saudi Arabia and one from Egypt—discuss respect for Islamic perspectives. Additionally, these references are merely general statements about respecting Islamic values. None of the guidelines refers to the documents and regulations released by Islamic medical entities such as the IOMS. This can be interpreted in two ways: either the authors of research ethics documents in the Middle Eastern countries see no contradiction between biomedical research ethics and Islam or, alternatively, there is no relation at all between the two and, therefore, no need to refer to Islam in any national document about biomedical research ethics. Moreover, none of the documents mentions any related fatwa, which indicates that fatwas are not well known or are not seen as important by national guideline makers [28].

However, theoretical agreement between ethical principles and cultural/religious values is insufficient. The interpretation and application of ethical principles may differ from one society to another. This is not limited to the situation in the Middle East, but also to other areas in the developing world, such as Africa [29]. Issues such as research on women, informed consent, and breaches of confidentiality require more attention in the guidelines and applications of research in the Middle East [25,28].

### Limitations

While the information in this study is based on the most recent data available, three restrictions on the data should be noted. First, analysis of and comparison between the contents of the guidelines among national documents and international documents—such as defining minors, incompetent persons, benefits, risks, and so forth—cannot be performed in a scientific manner because these guidelines are designed to provide concise and general ethical protections. Thus, there is no ability to estimate or analyze the meanings and dimensions behind the stated protections. Meaning is expected to be detailed in the manuals of the policies and procedures for research ethics guidelines, such as the newly published “Saudi Procedures List of the System of Ethics of Research on Living Creatures” (2011), which is the only manual related to research ethics available in the region (This version is in Arabic only. An English copy is expected to be published soon) [30].

Second, we do not include the ongoing efforts to establish guidelines in many countries, such as in Egypt, Jordan and Saudi Arabia, where it is expected that new guidelines will appear in the coming years.

Third, our paper does not report information on the process of ethical review at the national level or on what mechanisms are used to encourage adherence to regulations or guidelines.

## Conclusions

Recently, notable efforts to establish national research ethics guidelines have been made in many countries in the Middle East. However, compared with international documents, most of the research ethics documents in use in this region demonstrate numerous deficiencies. Many differences among the documents can be observed in the development, structure, content, and reference to international guidelines. Guidelines from GCC countries are more developed compared with guidelines from other countries in the Middle East. Greater efforts are required to develop better regulations and guidelines in most countries in the Middle East.

## Competing interests

The authors declare that they have no competing interests.

## Authors’ contributions

All authors participated in the design of the study. GA wrote the first draft. All authors have discussed all points in the first draft and in all subsequent drafts. All authors have read and approved the final manuscript.

## Pre-publication history

The pre-publication history for this paper can be accessed here:

http://www.biomedcentral.com/1472-6939/13/34/prepub
